# Effect of soy flour on nutritional, physicochemical, and sensory characteristics of gluten‐free bread

**DOI:** 10.1002/fsn3.411

**Published:** 2016-08-01

**Authors:** Maryam Taghdir, Seyed Mohammad Mazloomi, Naser Honar, Mojtaba Sepandi, Mahkameh Ashourpour, Musa Salehi

**Affiliations:** ^1^ Student Research CommitteeDepartment of Clinical NutritionSchool of Nutrition and Food SciencesShiraz University of Medical SciencesShirazIran; ^2^ Nutrition Research CenterDepartment of Food Hygiene and Quality ControlSchool of Nutrition and Food SciencesShiraz University of Medical SciencesShirazIran; ^3^ Pediatric Gastroenterology and Hepatology DepartmentShiraz University of Medical SciencesShirazIran; ^4^ Department of Epidemiology and BiostatisticsBaqyiatallah University of Medical SciencesTehranIran; ^5^ Nutrition Research CenterDepartment of Clinical NutritionSchool of Nutrition and Food SciencesShiraz University of Medical SciencesShirazIran

**Keywords:** Chemical properties, gluten‐free bread, physical properties, sensory characteristics, soy flour

## Abstract

The aim of this study was to assess the effect of soy flour on nutritional, physicochemical, and sensory characteristics of gluten‐free (GF) bread. In this study, corn flour was replaced with soy flour at different levels 5%, 10%, and 15% to produce a more nutritionally balanced GF bread. Physical and chemical properties, sensory evaluation and crust and crumb color were measured in bread samples. The results of evaluations showed that protein content of soy flour‐supplemented GF bread significantly increased from 9.8% to 12.9% as compared to control along with an increased in fat (3.3%–4.1%), fiber (0.29%– 0.38%), and ash (1.7%–2.2%) content. Moisture (27.9%–26.5%) and carbohydrate (58.3–52.3) content decreased with the incremental addition of soybean flour. The highest total score of sensory evaluation was for the bread sample containing 15% soybean flour. The evaluation of crust and crumb showed that bread samples with 15% soy flour were significantly darker than the other bread samples. In conclusion, adding higher levels of soybean flour into GF bread can improve bread quality, sensory characteristics, and nutritional properties of bread. Nutritional status in patients with celiac disease (CD) can be improved through the produce GF bread in this way.

## 1 Introduction

Presence of wheat gluten causes problems for consumers with celiac disease (CD: allergy to gluten), and prevalence of CD has caused an interest in gluten‐free (GF) products. An estimated up to 2% of the world population suffers from CD and a lifetime gluten‐free diet (GFD) is the only effective treatment for this disease (Bagolin Do Nascimento, Medeiros Rataichesck Fiates, Dos Anjos, & Teixeira, 2014; Ćurić, Novotni, Tušak, Bauman, & Gabrić, 2007). Glutenin and prolamin are the main fractions of gluten which provide viscosity, extensibility, elasticity, and cohesive properties of dough (Gujral & Rosell, 2004). So, it is difficult to produce an acceptable bakery product lacking gluten proteins.

GF dough usually has a soft consistency and is more sensitive to dough system collapse, which results in large holes at the bottom of crumbs. To improve structure, sensory aspects, and shelf life of bread in absence of gluten, recent researches have focused on adding dairy proteins and hydrocolloids to naturally GF flour. Although, available GF breads have still a dry crumbling crumb and poor mouth feel and flavor (Jeong, Kang, & Shin, 2013; Mccarthy, Gallagher, Gormley, Schober, & Arendt, 2005; Sanchez, Osella, & De La Torre, 2004).

Many GF products were not enriched and often were prepared with refined GF flour or starch (Thompson, 1999). GF food staffs, which have not been fortified, are poor sources of fiber, iron, folate, thiamine, riboflavin, niacin, and protein (Thompson, 1999). Enriched or fortified GF products improve the quality of GF diet (Jideani & Onwubali, 2009). Soybean could be an essential part of functional foods, as well as it could be used for enhancement of product quality (Ahmad et al., 2014). Soybean also contains up to 45% protein (Islam, Chowdhury, Islam, & Islam, 2007) with a digestibility value of 91.41% (Zhao et al., 2014) and as a good source of vitamins and mineral supplies adequate amount of different amino acids required for repairing the damaged body tissues. Soy consumption is associated with decrease in certain disease including diabetes, atherosclerosis, and cancer (Ahmad et al., 2014; Mohammadi Sartang, Mazloomi, Tanideh, & Rezaian Zadeh, 2015).

Soybean proteins include all the essential amino acids that are important for health. Soybean protein is about four times of wheat, six times of rice grain and it is also rich in Ca, P and Vitamins A, B, C, and D (Islam et al., 2007; Serrem, Kock, & Taylor, 2011). Fortified cereal with soy protein, especially when mixed with proper ratio, is one of the best sources of protein (Wadud, Abid, Ara, Kosar, & Shah, 2004). Soybean flour has been used to improve protein quality and shelf life of bread (Mohamed, Rayas‐Duarte, Shogren, & Sessa, 2006; Sanchez et al., 2004). Also, some studies have shown that adding soy flour (0.5%) to GF flour improves the quality of the bread (Sanchez, Osella, & Mdl, 2002). On the other hand, Iranian diet is mostly dependent on bread as major energy source (Rostami, Malekzadeh, Shahbazkhani, Akbari, & Catassi, 2004). The percentage of carbohydrate and fat in this type of diet has been evaluated 66% and 22% of total energy, respectively (Posner, Quatromoni, & Franz, 1994). Therefore, the main challenge for food scientists about GF products is production of high‐quality GF bread (Rostami et al., 2004). So, this study aimed to determine the effect of adding different percentages of soy flour on nutritional, physicochemical and sensory characteristics of GF bread to produce bread with optimum characteristics.

## 2 Materials and Methods

Soy flour (40% proteins, 19% lipid, and 8% moisture) was supplied by Soyan Toos Company, Iran. Cassava starch (0.3% protein, 0. 2% ash, and 9.3% moisture), corn starch (0.6% protein, 0.13% ash), corn flour (4.8% protein, 0.41% ash and 9.6% moisture), and rice flour (9.2% protein, 0.6% ash and 5.7% moisture) were obtained from market. Other ingredients were sodium caseinate (Iran Caseinate Company, Iran); fat (Sunflower oil); sugar; salt and yeast *Saccharomyces cerevisiae*.

### 2.1 Preparation of bread

Solid ingredients corn flour, 50, 45, 40, and 35%; corn starch, 10%; rice flour,13%; cassava starch, 8.5%; soy flour, 0, 5, 10, and 15%; fat, 7%; yeast, 2.5%; salt, 1%; sugar, 3% and sodium caseinate 5% were mixed with water (460 ml) at 400 rpm for 1 min and 600 rpm for 2 min. Dough was divided into 17 parts (60 gr), then proofed at about 27°C and 80% humidity for 15–20 min. Breads were baked at 225°C for 20 min with steam. The loaves were cool down for a minimum of 2 hr at 24°C before evaluation.

### 2.2 Methods of analysis

#### 2.2.1 Loaf specific volume

Loaf specific volume (SLV) is considered as one of the most important criteria in evaluating bread quality since it provides quantitative measurements of baking performance (Boye, Zare, & Pletch, 2010; Tronsmo, Færgestad, Schofield, & Magnus, 2003). SLV was expressed as the volume/mass ratio of bread. Weight (g) of bread was determined after cooling for 60 min according to the methods described in AACC (2000). Also, bread volume was determined using millet seed displacement method 1 hr after taking away from the oven as described by the Approved Methods of the AACC (2000) (Method No.10‐10‐B).

#### 2.2.2 Moisture content

Moisture content was determined after storage for 24 h at room temperature (25 ± 2°C) according to the method described in AOAC (2000).

#### 2.2.3 Ash content

The ash was determined by burning the known weights of the samples in a muffle furnace as recommended by the AACC (2000).

#### 2.2.4 Crude protein

The percentage of protein was determined by Kjeldahl method as recommended by the AOAC (1995). The conversion factor of nitrogen to protein was 6.25.

#### 2.2.5 Crude fat

The crude fat was determined by extracting a known weight of sample in petroleum ether (boiling point, 40–60°C) in a Soxhlet extractor (AACC, 2000).

#### 2.2.6 Crude fiber

Crude fiber was determined as recommended by the AACC (2000).

#### 2.2.7 Carbohydrate content

The available carbohydrate was measured by the difference method (the percent crude protein, fat, fiber, and ash minus percent dry matter) (FAO, 2003).

### 2.3 Sensory evaluation

Bread samples prepared by different levels of soy flour were evaluated by 30 taste‐testing panel judges comprising of workers with more than 10 years of experience in baking and teachers, scientific officers and students of the School of Nutrition and Food Sciences affiliated by Shiraz University of Medical Sciences. The bread samples were served as slices including the center points, at the same time. The panelists were requested to evaluate the bread on the basis of acceptance of its color, texture, taste, and overall quality on a 5‐point hedonic scale which ranged from 1 (dislike extremely) to 5 (like extremely) for each organoleptic characteristic.

### 2.4 Crust and crumb color

By a digital camera in a box with fluorescent light, the color characteristics of the sample bread loaf were determined. The photos were analyzed with the Adobe Photoshop software version 8 (Kazemi, Mazloomi, Hassanzadeh‐Rostami, & Akhlaghi, 2014). Crust color was measured on four different zones of the top of the loaf of bread. Crumb color was measured on four equidistant points to the center of each slice. The color parameters were determined in the Lab mode of the software where L* indicate lightness/darkness, a* indicate redness/greenness axis, and b* indicate yellowness/blueness axis (Kazemi et al., 2014).

### 2.5 Statistical analysis

The experiments were performed in a randomized design and performed at least in triplicates. Analysis of variance (ANOVA) and Kruskal–Wallis Test were used to study the differences between samples. Duncan's multiple range test (*p* < .05) was used to determine the significances within treatments. Statistical analysis of the data was performed using the SPSS software (SPSS, Inc., USA).

## 3 Results and Discussion

### 3.1 Physicochemical properties of gluten‐free bread with different levels of soy flour

Bread samples were prepared with 0, 5, 10, and 15% soy flour and subsequently compositions of the bread were determined and the results were presented in Table 1.

**Table 1:** Physicochemical properties of bread with different levels of soy flour

Samples	Moisture (%)	Protein (%)	Fat (%)	Ash (%)	Fiber (%)	Carbohydrate (%)	Specific Volume
(Control)	27.9 ± 1.4	9.8 ± 0.44^a^	3.3 ± 0.14	1.7 ± 0.25	0.29 ± 0.07	58.3 ± 3.2	2.7 ± 0.19^a^
5% soy flour	27.2 ± 2.3	10.7 ± 1.1^a^	3.6 ± 0.56	1.8 ± 0.24	0.31 ± 0.12	56.6 ± 1.7	2.6 ± 0.06^a^
10% soy flour	26.8 ± 0.10	11.5 ± 0.24^a^	3.8 ± 0.07	1.9 ± 0.11	0.35 ± 0.21	55.3 ± 2.2	2.5 ± 0.32^a^
15% soy flour	26.51 ± 2.6	12.9 ± 0.26^b^	4.1 ± 0.84	2.2 ± 0.26	0.38 ± 0.05	52.3 ± 1.6	1.6 ± 0.06^b^
*p*‐value^a^	N.S	0.03	N.S	N.S	N.S	N.S	0.01

Different letters in the same column indicate significant differences (*p* < .05).

a Over all *p*‐Value for Analysis of variance Test.

#### 3.1.1 Moisture and ash content

Although there was no significant difference in the moisture content, the highest moisture content was observed in control bread (27.9%) which is in agreement with the other studies (Banureka & Mahendran, 2011; Farzana & Mohajan, 2015; Olatidoye & Sobowale, 2011). The moisture content decreased gradually with the incremental addition of soy flour (27.9%–26.5%). This might be due to the fact that soy flour contain higher amount of solid matters with high emulsifying properties compared to corn flour. This findings show that the fortification of GF bread with soy flour could produce a more shelf stable bread due to its lower moisture content (Jimoh & Olatidoye, 2009).

It was seen that the highest ash content was in the sample containing 15% soy flour (2.2%) and the lowest in control bread (1.7%). The same as other studies, the ash content increased with increasing level of soy flour in the bread samples (Abioye, Ade‐Omowaye, Babarinde, & Adesigbin, 2011; Akpapunam, Badifu, & Etokudo, 1997; Awasthi, Siraj, Tripathi, & Tripathi, 2012; Farzana & Mohajan, 2015; Hegstad, 2008; Islam et al., 2007). Several studies have reported that the soy bean is rich in minerals (Onyeka & Dibia, 2002; Plahar, Okezie, & Gyato, 2003).

#### 3.1.2 SLV

In accordance with other studies (Islam et al., 2007; Sanchez et al., 2002), there was a reduction of SLV caused by soybean flour addition (Table 1). This difference was significant between bread samples with 15% soy flour and other bread samples. It gradually decreased with increasing level of soy flour in bread formulation (The results varied from 1.6 to 2.7 cc/g). Higher specific volume could be because of large bubbles that destroy crumb structure. Soy protein, as a water‐binding factor with stabilizing property which is unaffected during baking process, may modify this effect by preventing merger of bubbles in the crumb (Sanchez et al., 2002).

#### 3.1.3 Protein content

In line with other studies (Abioye et al., 2011; Ayo, Ayo, Popoola, Omosebi, & Joseph, 2014; Islam et al., 2007; Olaoye, Onilude, & Idowu, 2006), the protein content of different bread samples, from 9.8% to 12.9%, gradually increased with increasing level of soy flour as shown in Table 1. It may be due to the fact that the soy flour contain the higher amount of protein. This improvement is important because the protein contents of GF products are inadequate (Bagolin Do Nascimento et al., 2014). The main storage protein of soybean is globulins which contain almost 90% of soybean protein (Hou & Chang, 2004). Soy flour might increase the protein content of bread. This high protein content has a nutritional importance in most developing countries, where many people and especially children, can rarely intake adequate foods with high protein content because of the costs (Edem, Ayatse, & Itam, 2001). Also, some studies have shown that food proteins could influence on quality and functional properties of food goods, so soy protein may improve GF bread quality (Gerrard, 2002).

#### 3.1.4 Fat content

It was found that the fat content of soy bread samples were more than that of control bread sample and the fat content increased with the increasing level of soy flour. This is in agreement with the results of the other studies (Awasthi et al., 2012; Ayo et al., 2014; Banureka & Mahendran, 2011; Jimoh & Olatidoye, 2009; Olatidoye & Sobowale, 2011). The fat content of the GF bread changed from 3.3% to 4.1% with increase in soybean flour from 0% to 15% (Table 1). This is due to the fact that the fat content of soy flour is higher in comparison to corn flour (Abioye et al., 2011; Akpapunam et al., 1997; Islam et al., 2007). Soybean is an edible oil source with about 20%–24% fat content (Reddy, 2004). Like other vegetable, it is rich in unsaturated fat (61% polyunsaturated fat and 24% monounsaturated fat). Also, soybean is rich in polyunsaturated fatty acids such as linoleic and linolenic acid, which are necessary for human health (Hegstad, 2008).

#### 3.1.5 Crude fiber

In consistent with other studies (Ayo et al., 2014; Farzana & Mohajan, 2015; Ndife, Abdulraheem, & Zakari, 2011), crude fiber content was improved from 0.29% to 0.38% by rising the soy flour content from 0% to 15%. The crude fiber includes the cellulose components. The soy flour may contain higher amount of this type of fiber than that of corn flour.

#### 3.1.6 Carbohydrate content

Similar to other studies, the total carbohydrate content of soy flour free (Control sample) bread was higher (58.3%) than that of soy flour content bread samples (Abioye et al., 2011; Akpapunam et al., 1997; Awasthi et al., 2012; Islam et al., 2007; Jimoh & Olatidoye, 2009; Olatidoye & Sobowale, 2011). Soybean seeds contain 35% carbohydrates, which are comprised of digestible sugars, starch, and nondigestible oligosaccharides (Karr‐Lilienthal, Kadzere, Grieshop, & Fahey, 2005). The variations in carbohydrate content among the bread samples may result from the difference in the level of ash, fat, protein, and moisture content of corn and soy flours.

### 3.2 Sensory characteristics of GF bread with different levels of soy flour

The effect of soy flour on sensory characteristics of soy bread samples (color, taste, flavor, texture, and overall acceptability) were measured by the panel judges and the results are presented in Table 2.

**Table 2:** Sensory evaluation of the attributes scores in breads with different levels of soy flour

Samples	Taste	flavor	Color	Texture	Overall acceptability
50% Corn flour with 0% soy flour (Control)	4.15 ± 0.81	4.05 ± 0.826	4.25 ± 0.78	3.45 ± 0.51^a^	4.25 ± 0.78
45% Corn flour with 5% soy flour	4.17 ± 0.85	4.25 ± 0. 550	4.40 ± 0.786	3.3 ± 0.57^a^	3.95 ± 0.82
40% Corn flour with 10% soy flour	4.2 ± 0.83	4.10 ± 0. 718	4.50 ± 0.513	4.05 ± 0.94^b^	4.20 ± 0.83
35% Corn flour with 15% soy flour	4.35 ± 0.74	3.95 ± 0 .887	4.55 ± 0.754	4.1 ± 0.85^b^	4.45 ± 0.68
*p*‐value^a^	N.S	N.S	N.S	0.001	N.S

Different letters in the same column indicate significant differences (*p* < .05).

a Over all *p*‐Value for Kruskal–Wallis Test.

In this study, with regard to taste, texture, color, and overall acceptability, the sensory characteristics score of bread containing 15% soy flour, compared to 0, 5, and 10 percentage of soy flour, were found to be the highest.

The taste is the most important factor which affects the acceptability of an edible product (Banureka & Mahendran, 2011; Farzana & Mohajan, 2015). Although not significant, there was an increase in score for taste from 4.15 to 4.35 by increasing in the soy flour percentage.

The score for color of GF bread samples changed from 4.25 to 4.55. The highest score (4.55) was found for bread containing 15% soy flour. The score for color increased with the increase in the level of soy flour which was not significant. The color of the GF bread samples improved from creamy to brown. The darker color of GF bread samples with soy flour may be due to the presence of yellow pigment in the soybean flour and Maillard reaction during processing (Banureka & Mahendran, 2011; Olatidoye & Sobowale, 2011).

With the increase in substitution of soy flour to the GF bread samples, the crust texture increased from 3.45 to 4.1. The bread containing 15% soy flour had the highest score (4.1) and the bread containing 5% soy flour had the least score (3.3). The score of crust texture improved with the increase in the level of soy flour which was statistically significant (*p* = .001). It has been shown that appearance of bread is an important sensory parameter (Hoseney, 1994).

Flavor of GF bread samples decreased from 4.05 to 3.95 with increasing in the substitution of soybean flour. The GF bread containing 5% soy flour had the highest score (4.05) and the GF bread containing 15% soy flour had the least score (3.95). This may be due to the beany flavor of soy flour (Akubor & Ukwuru, 2003). Overall acceptability is one of the important factor in sensory evaluation (Banureka & Mahendran, 2011; Farzana & Mohajan, 2015). Bread containing 5% soy flour had the lowest overall acceptability (3.95 ± 0.82) and the highest overall acceptability was calculated for bread containing 15% soy flour (4.45 ± 0.68). This difference was not statistically significant.

At the 15% level of soy flour substitution, the bread had higher scores for all the sensory characteristics except flavor. Some studies have shown that addition of 10% or 15% soy flour to other flour produce acceptable products (bread or biscuit)(Awasthi et al., 2012; Banureka & Mahendran, 2011; Farzana & Mohajan, 2015; Jimoh & Olatidoye, 2009). Thus, incorporation of soy flour more than 15% did not produce acceptable products.

Color together with texture and aroma, contributes to consumer preference. It is influenced by physicochemical parameter of dough (Ahmad et al., 2014). GF breads often have low quality, undesired taste and flavor and poor crust and crumb characteristics (Thompson, 1999). Zarkadas et al. (2006) have reported that despite of increasing availability of GF foods in recent years, there is a difficulty in finding good‐quality GF foods for most of CD patients. Soybean flour has been used in bread in previous studies (Abioye et al., 2011; Akpapunam et al., 1997; Islam et al., 2007; Sanchez et al., 2004). Some authors have found that soy could improve the crumb, bread volume, and absorption properties of the bread (Moore, Schober, Dockery, & Arendt, 2004; Sanchez et al., 2004).

### 3.3 Crust and crumb color of gluten‐free bread with different levels of soy flour

Table 3 presents L*, a, and *b* values for breads. Considering crust and crumb color, a lower L* value indicated darkness, a* parameter indicated redness, whereas a higher b* value led to a higher yellowness. Bread sample with 15% soy flour had significantly lower L* values, showing darker than the other bread samples. Adding soy flour decreased L* value because of the flour color, and Maillard and caramelization reaction, which are affected by the reaction between amino acids and sugars and water distribution (Posner et al., 1994). Similar results were obtained by Zhao et al. (2014). Chattopadhyay, Raychaudhuri, & Chakraborty (2013) found that the visual color is directly related to acceptance and taste. GF breads usually tend to have a light crust color, so the darkening of the crust color due to soy addition is appropriate (Gujral & Rosell, 2004).

**Table 3:** Color parameters (Crust and Crumb) in breads with different levels of soy flour

Samples	Crust			Crumb		
	L	a	b	L	a	b
50% Corn flour with 0% soy flour (Control)	58.25 ± 2.63^a^	11.25 ± 0.95^a^	47.25 ± 0.95^a^	54.5 ± 1.3^b^	4 ± 0.8^a^	33.7 ± 1.26^b^
45% Corn flour with 5% soy flour	57.25 ± 2.9^a^	12.5 ± 1^a^	47.5 ± 1.3^a^	52.7 ± 1.26^a^	4.25 ± 1.5^a^	35.25 ± 3.8^b^
40% Corn flour with 10% soy flour	57.25 ± 2.63^a^	14.25 ± 1.5^a^	48 ± 1.4^a^	51.25 ± 1.7^a^	4.5 ± 1^a^	39.7 ± 3.5^a^
35% Corn flour with 15% soy flour	46.75 ± 1.5^b^	20.25 ± 0.95^b^	55.5 ± 1.9^b^	49.2 ± 0.9^c^	5.5 ± 0.57^a^	40.2 ± 0. 5^a^
*p*‐value^a^	0.0001	0.0001	0.0001	0.0001	N.S	0.011

Values with different letters within a column are significantly different.

a Over all *p*‐Value for Analysis of variance Test.

## 4 Conclusion

Adding soy flour can improve quality and nutritional properties of wheat bread (Islam et al., 2007). The results of this study showed that adding 15% soy flour to the GF bread formulation, improved bread quality, sensory characteristics, and nutritional properties of bread. Therefore, in order to prevent major CD complications, such as growth failure and weight loss, through a healthy diet, consumption GF bread containing 15% soy flour could be beneficial.

## Conflict of Interest

No potential conflict of interest relevant to this article was reported.
